# Exploring the influence of the gut microbiota and probiotics on health: a symposium report

**DOI:** 10.1017/S0007114514001275

**Published:** 2014-07

**Authors:** Linda V. Thomas, Theo Ockhuizen, Kaori Suzuki

**Affiliations:** 1 Yakult UK Limited, Odyssey Business Park, West End Road, South Ruislip, MiddlesexHA4 6QQ, UK; 2 Nutricom Consultancy, Dorpsdijk 10, 4156 AKRumpt, The Netherlands; 3 Yakult Europe B.V., Schutsluisweg 1, 1332 ENAlmere, The Netherlands

**Keywords:** Intestinal microbiota, Probiotics, Immune System, Metabolism, Metagenomics, Inflammation, Disease

## Abstract

The present report describes the presentations delivered at the 7th International Yakult Symposium, ‘The Intestinal Microbiota and Probiotics: Exploiting Their Influence on Health’, in London on 22–23 April 2013. The following two themes associated with health risks were covered: (1) the impact of age and diet on the gut microbiota and (2) the gut microbiota's interaction with the host. The strong influence of the maternal gut microbiota on neonatal colonisation was reported, as well as rapid changes in the gut microbiome of older people who move from community living to residential care. The effects of dietary changes on gut metabolism were described and the potential influence of inter-individual microbiota differences was noted, in particular the presence/absence of keystone species involved in butyrate metabolism. Several speakers highlighted the association between certain metabolic disorders and imbalanced or less diverse microbiota. Data from metagenomic analyses and novel techniques (including an *ex vivo* human mucosa model) provided new insights into the microbiota's influence on coeliac, obesity-related and inflammatory diseases, as well as the potential of probiotics. *Akkermansia muciniphila* and *Faecalibacterium prausnitzii* were suggested as targets for intervention. Host–microbiota interactions were explored in the context of gut barrier function, pathogenic bacteria recognition, and the ability of the immune system to induce either tolerogenic or inflammatory responses. There was speculation that the gut microbiota should be considered a separate organ, and whether analysis of an individual's microbiota could be useful in identifying their disease risk and/or therapy; however, more research is needed into specific diseases, different population groups and microbial interventions including probiotics.

The 7th International Yakult Symposium, ‘The Intestinal Microbiota and Probiotics: Exploiting Their Influence on Health’, was held in London on 22–23 April 2013. Over 300 scientists from twenty-three different countries attended, representing clinical and academic researchers from a wide range of disciplines.

Dr Kenji Oishi (Yakult Honsha European Research Centre, Ghent, Belgium) described research into the microbial colonisation of the gastrointestinal (GI) tract immediately after birth, which can have lifetime consequences if an aberrant microbiota predisposes to disease later in life. Professor Paul O'Toole (University College Cork, Ireland) described studies on the effects of diet, residence and antibiotic use on the gut microbiota and markers of health risk in older people.

Several talks focused on the metabolic activity of the intestinal microbiota. Professor Harry Flint (University of Aberdeen) discussed butyrate metabolism in the colon and how this is affected by diet. Professor Joël Doré (National Institute for Agricultural Research, France) gave an update on human faecal metagenomic research, which has collected an extensive gene repertoire representative of the functional potential of the human intestinal microbiome, and associated dysbiosis with certain diseases. Professor Patrice D. Cani (Université catholique de Louvain, Belgium) examined the association between the gut microbiota and obesity-related disorders, and the effects of metabolic endotoxaemia. Professor Fredrik Bäckhed (University of Gothenburg, Sweden) described metagenomic studies in different parts of the world; some have indicated an association between dysbiosis and the risk of diabetes.

In a series of talks relating to infection and inflammation, the gut barrier and its role in GI and hepatic disease was covered by Professor Stephan Bischoff (University of Hohenheim, Germany); Professor Julie-Stefanie Frick (University of Tübingen, Germany) described how the host recognises pathogenic bacteria; and Professor Hiroshi Kiyono (The University of Tokyo, Japan) discussed how the intestinal microbiota influences the mucosal immune system to respond as tolerance or defence. This theme was continued by Professor Maria Rescigno (European Institute of Oncology, Italy) who described model systems for the preclinical assessment of probiotics for inflammatory bowel disease (IBD). IBD was also discussed by Professor Jerry Wells (Wageningen University, The Netherlands) who focussed on research into *Faecalibacterium prausnitzii*, and by Dr Ailsa Hart (St Mark's Hospital, London, UK) who reviewed clinical trials investigating the outcome of the modulation of the gut microbiota. Finally, Professor Yolanda Sanz (National Research Council, Spain) outlined the latest research into the gut microbiota and coeliac disease.

Further explanation of terms used in the present report is given in Table 1.

**Table 1:** Further explanation of some of the terms used in the present report

Terms	Explanation
16S rRNA gene sequencing	The 16S ribosomal RNA gene, present in all bacteria, is used as a genetic marker to study bacterial phylogeny and taxonomy. Because its function has remained unchanged, any change in sequencing is a measure of time and evolutionary relatedness
Cannabinoid system	A lipid signalling system with important regulatory functions in the brain and autonomic nervous system, as well as in the immune system. It consists of endocannabinoids (cannabis-like molecules produced in the body) and a family of G-protein receptors
Claudins	Proteins that are key components of tight junctions, which promote cell-to-cell adhesion and regulate paracellular transport. At least twenty-four family proteins have been described
Cytokines	Proteins produced by immune cells that affect the behaviour of other cells. Cytokines produced by lymphocytes are called interleukins
Defensins	Cationic small peptides produced by neutrophils and Paneth cells in the gut, which have antimicrobial activity
Dendritic cells	Specialised antigen-presenting mononuclear monocytes that control and direct both innate and acquired immune responses
Gnotobiotic	A term used to describe animals having a known set of microbes in and on the body, such as in the gut. Gnotobiotic animals are developed from neonates born and raised in sterile conditions by inoculation with known specific micro-organisms. This word is derived from the Greek for ‘gnostos’ meaning known and ‘bios’ meaning life
Lipopolysaccharide	A major component of the outer cell membrane of Gram-negative bacteria; it is an endotoxin
Metagenomics	The genomic analysis of micro-organisms by direct extraction and cloning or direct sequencing of all DNA recovered from a specific environment, such as the human body, containing a mixed community of micro-organisms
Microbiome	All the DNA, or genomes, of all the micro-organisms present in one environment, such as the human body
Microbiota	The community of micro-organisms populating one defined environment, such as the gut
Mucin	Large glycoproteins that are the prime constituent of mucus. They are produced by goblet cells in the gut
PCR	A fast and inexpensive laboratory technique to make millions of copies of a DNA sequence from just one or a few pieces of DNA. Once the DNA has been amplified, it can be mapped, sequenced and fingerprinted
Splanchnic hypoperfusion	Decreased blood flow to the internal organs
Tight junctions	Components of intestinal epithelial cells that connect neighbouring cell membranes to form a virtually impermeable barrier and to regulate diffusion of ions and solutes between adjacent cells

## The gut microbiota: impact of age, diet and contribution to disease

### How colonisation of neonates is influenced by the maternal gut microbiota

Dr Oishi explained that intestinal colonisation occurs immediately after birth, with dramatic changes in the microbiota composition in the first few days of life. This has been shown by several studies, including the study by Tsuji *et al.* ^(^ ^1^ ^)^ of 166 healthy Japanese neonates, which used quantitative RT-PCR to analyse their faecal microbiota from the 1st day after birth until the age of 3 years. Over the first 30 d, analysis of anaerobic bacteria (obligate and facultative) showed an initial predominance of Enterobacteriaceae, followed later by a rise in the numbers of *Bifidobacterium* spp. and *Clostridium* groups.

Several external factors influence the colonisation sequence and eventual profile of the intestinal microbiota in early infancy. A large study in The Netherlands^(^ ^2^ ^)^, for example, found that babies born by caesarean section had lower numbers of bifidobacteria and *Bacteroides* compared with those born vaginally, and were more likely to be colonised with *Clostridium difficile*. The risk of *C. difficile* colonisation increased when the babies stayed longer in hospital.

As well as horizontal transmission from external sources (surroundings and diet), colonising microbes can also be transmitted vertically, i.e. from the mother to the baby. A study in an obstetrics department in Venezuela^(^ ^3^ ^)^ used multiplexed 16S ribosomal RNA gene pyrosequencing to analyse samples from different sites on mothers 1 h before delivery, and from the neonates (and meconium) immediately after delivery and within 24 h. Vaginally born babies acquired a microbial profile similar to the maternal vagina, usually dominated by *Lactobacillus* and *Prevotella* spp. In contrast, the microbial profile of babies born by caesarean section was more representative of that of the maternal skin, with *Staphylococcus* and *Corynebacterium* spp. being detected.

However, recent research^(^ ^4^ ^)^ has indicated that each individual may have a unique metagenomic genotype. It is very important that the gut microbiota is characterised not just at the level of species or phyla but also at the strain level. Analyses at species level are not sensitive enough to track the transfer of individual strains from the mother to the baby, so Dr Oishi's group used a more sensitive method: multi-locus sequence typing – a powerful and precise genotyping technique for characterising and classifying bacterial strains. They examined bifidobacteria isolated by culture from the faeces of mothers before delivery, and from their babies (meconium and faeces at days 3, 7, 30 and 90 after birth)^(^ ^5^ ^)^. More than 2500 strains were isolated from the mother/baby pairs (eighty-two vaginal deliveries; twenty-nine caesarean deliveries). Specific analysis of the strains of *Bifidobacterium* *longum* subsp. *longum* from the vaginal-delivery group showed that certain strains, previously predominant and stable in the pregnant mothers, were transferred to their babies soon after birth and then colonised their intestines. Interestingly, each transmitted strain was unique to its own cluster and to a particular mother–neonate pair. Similar transmission of the strains of *Bifidobacterium adolescentis*, *Bifidobacterium bifidum*, *Bifidobacterium catenulatum* and *Bifidobacterium pseudocatenulatum* was shown. No such transmission from the mother to the baby was observed in the caesarean-delivery group.

Dr Oishi concluded by emphasising how important it is for women to have a balanced intestinal microbiota during pregnancy. In the question and answer session that followed, there was a debate whether, based on the above findings, women with IBD should be advised to have caesarean delivery to avoid transfer of what could be an ‘unhealthy’ microbiota.

### Correlations between diet, health and the gut microbiota in older persons

Changes in the gut microbiota also occur in later life. Professor O'Toole described how the advent of culture-independent techniques for microbiota analysis has given further insight into the nature of these changes and their health implications. However, there have been contradictory results: for example, different genera and species found to be depleted or abundant in older people compared with younger people, and country-specific differences^(^ ^6^ ^,^ ^7^ ^)^.

The objective of the ELDERMET (http://eldermet.ucc.ie/) project, launched in Ireland in 2007, was to perform a detailed study of 500 people aged over 65 years, to investigate any associations between diet, the gut microbiota and health in a clinically well-phenotyped group. Over 6 months, faecal, blood, urine and saliva samples were analysed, and anthropometric measurements and other indicators of physical and mental health recorded.

Initial studies focussed on the choice and optimisation of molecular techniques^(^ ^8^ ^–^ ^10^ ^)^, but in 2011, the first major findings were published^(^ ^11^ ^)^. Baseline analysis of 161 subjects showed distinct differences between the core microbiota and its aggregate composition in older subjects compared with younger subjects. Significant inter-individual variations were observed at the phylum (e.g. ratio of Bacteroidetes:Firmicutes) and genus levels (e.g. *Ruminococcus* and *Faecalibacterium*).

The next trial^(^ ^12^ ^)^ analysed 178 subjects not receiving antibiotics, from whom dietary intake information was collected using a FFQ and who were stratified by where they lived (long-term residential care, rehabilitation hospital care for < 6 weeks, attending an outpatient day hospital or living in the community). It found distinct differences in the microbiota. The microbial profile of those living in the community or attending day hospital was similar to that of younger adults, whereas the profile of those in institutional care was notably different and less diverse. For example, genera such as *Roseburia*, *Coprococcus* and *Hydrogenoanaerobacterium* were more abundant in the faeces of community dwellers, whereas genera such as *Parabacteroides*, *Eubacterium* and *Subdoligranulum* were more abundant in residential dwellers. The latter also had a higher ratio of Bacteroidetes:Firmicutes.

Dietary analysis revealed a worryingly low intake of fruit and vegetables for elderly subjects in long-stay care institutions, whose diet seemed to be high in fat and low in fibre, heavy with starchy and sugary foods with a high energy value. People living in the community tended to have a more healthy diet: intake of more fibre, less red meat and more oily fish. Microbial diversity correlated with these dietary differences, and also with place of residence. The dietary intake changed within 2 weeks of moving from living in the community to institutional care, whereas changes in the gut microbiota profile were not evident for at least a year. Diet appears to be the key driver of change, influencing the composition of the microbiota, which in turn influences health.

The results of culture-dependent methods showed that faecal bifidobacteria levels were significantly reduced following the use of antibiotics, while the levels of lactobacilli and Enterobacteriaceae did not change. (The antibiotics administered included nucleic acid synthesis inhibitors, cell envelope antibiotics, protein synthesis inhibitors and others.)^(^ ^13^ ^)^ However, the drop in the levels of bifidobacteria was most marked in subjects from long-stay care institutions, suggesting that people with a less diverse gut microbiota are more susceptible to antibiotic-associated dysbiosis. A correlation was also observed between residential location and the carriage rate of *C. difficile* ^(^ ^14^ ^)^: 1·6 % for subjects living in the community; 9·5 % in outpatient settings; as high as 21 % for hospital patients (short and long terms). The gut microbiota profile of asymptomatic carriers was similar to the profile of those negative for *C. difficile*; in contrast, a reduced microbial diversity was observed in patients diagnosed with *C. difficile*-associated diarrhoea at the time of sampling and from whom the hypervirulent strain R027 was isolated.

Subjects could also be clustered by their faecal metabolite profile (analysed by NMR spectroscopy of faecal water), with, for example, higher levels of glucose, glycine and lipids being found for long-stay dwellers. Shotgun metagenomic sequencing showed higher gene counts and coverage for SCFA (e.g. butyrate, acetate and propionate) in the community-dwelling subjects, which correlated with their more diverse microbial profile^(^ ^12^ ^)^. This was the first indication of an association between diet, the gut microbiota and health status in these elderly people. Immune markers (IL-6, IL-8, IL-10, TNF-α and C-reactive protein) indicated a trend for a greater degree of inflammation for subjects in long-stay care institutions, although this was predictable because of their overall health profile. Data acquired from a battery of health and clinical tests showed an association between the microbiota composition and health status. Measures of independence and frailty correlated with faecal metabolome in twenty-seven subjects.

As the microbiota is driven by diet and the microbiota profile correlates with health status, the obvious next step would be to modulate or improve the health status of older people by programming the microbiota through dietary intervention, such as with the Mediterranean diet, probiotics and prebiotics. This will be the focus of NU-AGE, a €9 million project in Europe.

### Metabolic activity of the intestinal microbiota: effect of diet

Most of the human gut microbiota is present in the dense anaerobic communities of the large intestine where both diet- and host-derived energy sources are utilised for growth, predominantly through fermentative metabolism, explained Professor Flint. Major metabolic products are SCFA, which have an impact on the host in several ways (e.g. stimulation of host receptors that influence hormones and inflammation; lipogenesis by acetate; gluconeogenesis by propionate), although SCFA can be toxic at high concentrations. Butyrate has many important and protective functions, being an energy source for colonic epithelial cells and a regulator of mucosal gene expression, differentiation and apoptosis. It may also protect against colorectal cancer and colitis. The importance of butyrate in the gut was demonstrated in an analysis of stool samples from six overweight men on strictly controlled diets. More than 320 phylotypes were detected, and 25 % of cultured species accounted for approximately 50 % of the 16S ribosomal RNA sequences. Approximately 30 % of the dominant bacterial species could produce butyrate; these were *F. prausnitzii*, *Eubacterium rectale*, *Eubacterium hallii*, *Anaerostipes hadrus*, *Roseburia faecis*, *Subdoligranulum variabile*, *Roseburia inulinivorans* and two new species^(^ ^15^ ^,^ ^16^ ^)^.

Butyrate metabolism can proceed via one of two pathways: the relatively uncommon butyrate kinase route of *Coprococcus* spp. and the more common pathway involving butyryl-CoA: acetate-CoA transferase^(^ ^17^ ^)^. The latter group of bacteria fall into three main groups based on sequence analysis of the enzyme specific to their pathway: flagellated starch utilisers (e.g. *E. rectale* and *Roseburia* spp.); lactate utilisers (e.g. *E. hallii* and *Anaerostipes* spp.); *F. prausnitzii* ^(^ ^18^ ^,^ ^19^ ^)^. To investigate the effect of diet on butyrate-producing bacteria, a group of obese volunteers were put on different ‘Atkins-type’ diet for 4 weeks (a maintenance diet, a high-protein/medium-carbohydrate diet or a high-protein/low-carbohydrate diet). Although the subjects tended to lose weight on high-protein diets, analysis of their faecal microbiota and metabolites showed changes likely to be detrimental to colonic health: for example, reductions in SCFA, especially butyrate, which corresponded to a reduction in the *Roseburia*/*E. rectale* group. The high-protein diet also increased the proportions of branched-chain fatty acids and the concentrations of phenylacetic acid and *N*-nitroso compounds^(^ ^20^ ^,^ ^21^ ^)^. Experiments using mixed human faecal microbial communities in anaerobic continuous culture fermenters showed a similar but even more dramatic response to the changes in pH and peptide levels^(^ ^22^ ^,^ ^23^ ^)^. Due to cross-feeding between species in the gut, dietary intake affects both the metabolic pathways and the community structure of the intestinal microbiota.

Butyrate-producing bacteria in the gut are a diverse group of strains that are all sensitive to oxygen, although *F. prausnitzii* inhabits a unique niche within the gut mucus due to its ability to grow at the oxic–anoxic interface through an extracellular electron shuttle^(^ ^24^ ^,^ ^25^ ^)^. Some species show further metabolic diversity: for example, *E. hallii*, *A. hadrus* and *Anaerostipes caccae* are uniquely able to form butyrate from lactate and acetate, and contribute to inter-species cross-feeding of lactate^(^ ^18^ ^)^. *R. inulinivorans* can grow on glucose, starch or inulin to produce butyrate, but one strain can also grow on host-derived fucose, producing propionate and propanol as additional products^(^ ^26^ ^)^.

The core gene categories derived from metagenomics display less inter-individual variation than the phylogenetic groups of the human microbiota; thus, it might be tempting to simplify analysis by ignoring phylogeny. Professor Flint cautioned against this temptation: phylogeny gives much information about the associations between functions. In a recent cross-over trial in obese males, volunteers were given 3-weekly periods of three different diets: a diet high in NSP; a diet high in resistant starch; a weight-loss diet. The dietary changes correlated with clear changes in faecal metabolites and the relative abundance of the dominant phylotypes. *Ruminococcus bromii* and *E. rectale*, two species dominant in the gut, were particularly stimulated by increased resistant starch in the diet, and *Ruminococcus* spp., in particular, responded rapidly when the diet changed^(^ ^15^ ^)^. A decrease in species diversity was observed when subjects were on the resistant starch diet compared with the diet high in NSP. The sequence dataset as a whole showed a tendency for samples to cluster by individual rather than by diet. For example, two volunteers had markedly reduced ability to digest resistant starch, and this correlated with low levels of *R. bromii* and related species (*Ruminococcus* clostridial cluster IV spp.). *R. bromii* has now been identified as a keystone species with an exceptional ability to colonise and degrade starch in the human colon^(^ ^27^ ^,^ ^28^ ^)^.

The gut is not a homogeneous environment but has many different microenvironments^(^ ^16^ ^)^. Many of the substrates that act as nutrients for the microbiota are insoluble, for example, mucin secreted by the host, and dietary plant fibre. Analysis of the microbial communities in the liquid and particulate fractions of human faecal samples has shown that *R. bromii* and related species preferentially associate with insoluble fibre particles, whereas Bacteroidetes tend to partition in the liquid phase^(^ ^29^ ^)^.

Some aromatic compounds (such as phenylacetic acid) appear to be derived mainly from aromatic amino acids^(^ ^30^ ^)^. However, the majority are of plant origin, often being released by microbial hydrolases from glycosides present in the plant or of conjugates (e.g. glucuronides) formed in the liver. Bacterial β-glucuronidase in the human colon is important in cleaving such liver conjugates and xenobiotics. Obese volunteers on the high-protein/moderate-carbohydrate weight-loss diet showed an increase in faecal bacterial β-glucuronidase activity. Genes for this enzyme are unevenly distributed within the colonic microbiota; this may also be true for activities involved in transforming phenolic compounds released from plant fibre^(^ ^31^ ^)^.

Professor Flint gave the following conclusions. Dietary intake has a major impact on metabolites of microbial origin, partly because the diet causes the intestinal microbiota profile to change. There are important inter-individual variations in gut composition that influence the response to the diet, and perhaps also influence health. The presence of keystone species in the colon may determine an individual's ability to ferment insoluble substrates; there could be major consequences if such species are absent. Analyses based on functional groups can remove ‘noise’ and simplify system modelling and monitoring, but phylogenetic details remain important.

### Disease states associated with dysbiosis and low microbial diversity

Professor Doré explained that molecular analyses of the intestinal microbiota have shown that approximately 70 % of its dominant species have yet to be cultured. Over fifty phyla are represented, but only a few are dominant: Bacteroidetes; Actinobacteria; Firmicutes^(^ ^32^ ^)^. Single-gene 16S ribosomal DNA sequence-based approaches show that the gut microbiota has considerable species diversity; however, there is a core microbiota composed of only a few but prevalent species, which is resistant and resilient to change, thus important in maintaining homeostasis^(^ ^33^ ^)^.

Several strands of research suggest that a gut microbiota with low diversity may have negative consequences for health. Exposure to low bacterial diversity in the first few days of life, for example, prevents or delays maturation of the mucosal immune system, increasing the risk of an aberrant immune response and allergic disease (the hygiene hypothesis)^(^ ^34^ ^,^ ^35^ ^)^. Comparative analysis of the faecal microbiome of three cohorts (healthy Amerindians from the Amazonas of Venezuela, residents of rural Malawian communities and inhabitants of a US metropolitan area) found little differences up to the age of 3 years, but from then on, the microbial profile of the US group was clearly different, becoming much less diverse with fewer species^(^ ^36^ ^)^. If low microbial diversity is a health risk, then we need to understand why.

The human intestinal microbiome, representative of 10^13^ to 10^14^ microbes, has at least 100 times more genes than its host, which is why metagenomic analysis (which looks at the combined genomes of all dominant microbes within a given ecosystem) is an invaluable tool for investigating the association between the commensal gut microbiota (and its diversity) and disease risk^(^ ^37^ ^)^. Metagenomics involves extracting the DNA from the bacterial fraction of faeces, applying whole-genome shotgun sequencing to build a reference gene catalogue and recording gene counts^(^ ^38^ ^,^ ^39^ ^)^. The development of such techniques has led to several international human microbiome projects, for example, the MetaHIT project in the European Union and China (led by Professor S. D. Ehrlich), the MicroObes project in France (led by Professor Doré) and the Meta-GUT project in China (led by Professor Liping Zhao)^(^ ^40^ ^)^. Researchers from the MetaHIT project conducted deep sequencing of total DNA from faecal samples of 124 European people. A catalogue of 3·3 million genes was established, which showed that each person carries an average of about 540 000 genes. Subjects shared a core microbiome: about 50 % of each individual's genes were shared by at least 50 % of other individuals. Yet 2·4 million rare genes were also found, shared by less than 20 % of the subjects^(^ ^41^ ^)^. In an attempt to characterise the profile of an ‘average’ human intestinal microbiota, the researchers were able to group the microbiomes into three assemblages of gene and microbial taxa, termed enterotypes^(^ ^42^ ^)^. These shared specific traits but were independent of geographic origin, age, sex, etc. Individual-specific strains appear to be relatively stable, suggesting that individuals have a unique metagenomic genotype^(^ ^4^ ^)^. Using quantitative metagenomics, the human microbiome was shown to have a range of gene counts and different marker species were identified that indicated either low (e.g. *Bacteroides*) or high (e.g. *F. prausnitzii*) gene counts.

Metagenomic signatures of dysbiosis have been reported for certain immune-mediated diseases. For example, reduced abundance and diversity of Firmicutes has been observed in patients with Crohn's disease, and a reduced number of one species of this phylum, *F. prausnitzii* (an indicator of a high gene count microbiota), was associated with an increased postoperative risk of recurrence of the disease^(^ ^43^ ^)^. The association between Crohn's disease and low counts of *F. prausnitzii* has been observed in several other studies; low counts of *Subdoligranulum, Roseburia, Bifidobacterium* and other species have also been associated with this disease.^(^ ^44^ ^)^. A low gene count microbiota and low abundance of *F. prausnitzii* have both been associated with a high rate of ulcerative colitis (UC) relapse. A current human intervention study is investigating whether low gene counts in UC will predict whether a patient would respond to microbiota stabilisation by probiotic intervention.

Professor Doré described new data that indicate an association between low gene count and an increased risk of adiposity, insulin resistance, high blood lipid levels and inflammation^(^ ^45^ ^,^ ^46^ ^)^. Furthermore, people with low gene counts respond less well to nutritional intervention (e.g. low fat, high protein, low-glycaemic index carbohydrates, fibres from fruit and vegetables). Other diseases associated with low species richness of the intestinal microbiota include type 1 diabetes, type 2 diabetes, coeliac disease, allergy, autism, *C. difficile* infection and cystic fibrosis^(^ ^47^ ^)^.

A study comparing biopsies from the sigmoid colon of UC patients with those from their healthy twins found a difference in the microbial profile, as well as indications of a loss of interaction between the transcriptional profile of the mucosal epithelium and the colonic microbiota in UC patients^(^ ^48^ ^)^. This begs the following question: is microbial dysbiosis the cause or effect of this disease? If there is a vicious circle between altered intestinal ecology and altered physiology, can this be broken by modulating the intestinal microbiota? Functional metagenomics, a high-throughput screening method for metagenomic clone libraries, is being used to investigate the interactions between candidate probiotic bacteria and intestinal epithelial cells. To date, publications have reported modulation of immune functions, of epithelial cell turnover and of cellular metabolism^(^ ^49^ ^–^ ^53^ ^)^.

The gut microbiota should be considered as a separate organ of the host, argued Professor Doré, because it has unique functionalities that protect its host. It intimately interacts with food and human cells, may be aberrant in many diseases and may provide biomarkers that can be used to predict disease risk. Professor Doré stressed that alternative stable states of gut microecology may be associated with immune-mediated disease conditions, and that reduced microbial diversity is a robust indicator of altered intestinal ecology and physiology. Whether cause or effect, reduced microbial diversity contributes to the prolongation of chronic conditions, with altered crosstalk between the gut and its microbiota. Functional metagenomics offers a new window into this. Microbiome stratification is a promising tool that could be used to work towards personalised medicine, diagnosis and intervention. The latter may involve modulation of the microbiota, by means of diet, probiotics and/or prebiotics, to try to restore normality.

### Microbial targets for intervention

Obesity is characterised by a cluster of metabolic diseases (insulin resistance, glucose intolerance, hyperinsulinaemia, impaired fasting glycaemia, type 2 diabetes, complex dyslipidaemia, fibrinolysis disorder, endothelial dysfunction, hypertension and atherosclerosis). Professor Cani pointed out that these are all clinical disorders associated with low-grade inflammation.

For the last 15 years, he has been investigating how the intestinal microbiota interacts with nutrients and host biology to control obesity and its associated disorders. His group has shown that high fat feeding in mice induces a low-grade inflammation, and metabolic disease is associated with reduced intestinal bifidobacteria and increased plasma levels of endotoxin (endotoxaemia)^(^ ^54^ ^,^ ^55^ ^)^. Taken together, these findings indicate that endotoxin is a trigger factor for metabolic inflammation and insulin resistance^(^ ^56^ ^)^. Changes in the gut microbiota control this process by a mechanism that affects gut barrier function and increases intestinal permeability, which may involve the disruption of tight junctions^(^ ^57^ ^,^ ^58^ ^)^. Reducing endotoxin leakage from the gut into the bloodstream, perhaps by modulation of the gut microbiota, was suggested as a target in the strategy to reduce metabolic disease.

More than 18 years ago, Gibson & Roberfroid^(^ ^59^ ^)^ introduced the concept of prebiotics: dietary non-digestible oligosaccharides that promote the growth of beneficial bacteria already present in the human colon. Increased satiety and reduced feelings of hunger are two of the many targets for prebiotic-induced modulation of the gut microbiota^(^ ^60^ ^)^. For example, animal studies have shown that prebiotics reduce plasma endotoxin levels and decrease hepatic expression of inflammatory and oxidative stress markers. These beneficial changes were linked to an increase in glucagon-like peptide-2 (GLP-2) production; a GLP-2 antagonist prevented most of the prebiotic effects^(^ ^58^ ^)^. Professor Cani suggested that gut peptides such as this could be another target in the efforts to reduce metabolic disease.

The endocannabinoid (eCB) system is a lipid signalling system, composed of cannabis-like substances that are endogenous bioactive lipids (e.g. anandamide and 2-arachidonoylglycerol) that bind and activate specific G-protein-coupled receptors (the cannabinoid receptors CB1 and CB2) in the brain, affecting many different functions. Outside the brain, this system influences the autonomic nervous system, the immune system, GI functions and the microcirculation. An increased eCB system tone is observed in obesity, so this could be a target for investigation. In animal studies, blocking the CB1 receptor abolishes the low-grade inflammation associated with obesity^(^ ^61^ ^)^. In prebiotic experiments, the gut microbiota was shown to modulate the eCB system tone, which thus regulated gut permeability, plasma endotoxin levels as well as adipogenesis^(^ ^61^ ^)^.

Professor Cani also described prebiotic-induced changes in obesity-associated symptoms, including reduction of metabolic endotoxaemia, fat mass development, insulin resistance and gut permeability. Oligofructose feeding does not just change bifidobacteria numbers; it significantly changes more than 100 bacterial taxa, of which sixteen have been shown to increase or decrease by more than one logarithm^(^ ^62^ ^)^. Genetically and high-fat diet-induced obese and diabetic mice have much lower levels of *Akkermansia muciniphila*, but their levels could be restored with prebiotic intervention. This was surprising because this is a Gram-negative species (therefore a source of endotoxin) and prebiotics have been shown to reduce endotoxaemia. *A. muciniphila* levels also inversely correlated with fat mass, body weight, metabolic endotoxaemia and markers of inflammation. The administration of *A. muciniphila* to high-fat diet-induced obese mice restored their gut barrier function and increased the thickness of the mucus layer; this was again surprising as *A. muciniphila* degrades mucin. *A. muciniphila* administration also reduced obesity in these mice, despite no change in their diet and no fat malabsorption. A change in the intestinal eCB system tone was also observed, with an associated reduction in inflammation and increase in GLP-1 production. The mechanisms underlying these effects are not yet understood, although it appears that live bacteria are necessary and that *A. muciniphila* controls RegIIIγ expression in the colon^(^ ^63^ ^)^.

The take-home messages from Professor Cani were as follows: the gut microbiota contributes to energy homeostasis; bacterial compounds contribute to low-grade inflammation; gut permeability is a feature of obesity and type 2 diabetes. Prebiotics are powerful tools that can be used to investigate novel targets in tackling obesity, such as GLP-1/2, eCB and *A. muciniphila.* *A. muciniphila* may be either a new key player or even a team leader in the gut microbiota's influence in protecting against obesity-related disease. He stressed again that the presence of a mucin-degrading species in the gut does not necessarily mean a reduced thickness of the mucus layer. Crosstalk between *A. muciniphila* and cells of the intestinal epithelium and immune system can lead to increased production of mucus. Perhaps this bacterial species tells the host that it will help provide protection against invading pathogens if it is provided with more of its food source, i.e. mucus^(^ ^64^ ^)^.

### Type 2 diabetes: bacterial modulation of host metabolism

Professor Bäckhed explained how the spectrum of most common disease risks has shifted over the last 60 years, moving from infectious diseases to mainly those that are autoimmune or obesity-related, which explains the intensity of research into the influence of the gut microbiota on obesity and obesity-related disorders^(^ ^65^ ^–^ ^68^ ^)^.

A metagenomic study in China showed there are differences between the gut microbiota of healthy people and people with type 2 diabetes; for example, the diabetic patients had increased numbers of *A. muciniphila* ^(^ ^69^ ^)^. These interesting results have raised several questions: were the findings specific to this population and what was the role of medication, sex, etc. on the metagenome? To shed further light, during 2001–2003, Professor Bäckhed's group initiated a large prospective study, by inviting all women aged 64 years in Gothenburg to take part in a screening exercise. From this, a cohort was identified who had normal, impaired or diabetic glucose control. In 2007–2009, these women were re-examined and a subgroup was randomly selected for a metagenomic study of faecal samples^(^ ^70^ ^)^. Genomic DNA was extracted and shotgun sequenced, and the data were analysed using a bioinformatics pipeline (Metagenomic Data Utilization and Analysis)^(^ ^71^ ^)^. Sets of genes with high correlation were clustered (metagenomic clusters, MGC), which allowed previously unsequenced DNA to be included in the analysis. The researchers then investigated the association between species, MGC and clinical biomarkers. Compositional and functional differences were observed in those with type 2 diabetes. Enough data were collected to enable the development of a mathematical model that could classify type 2 diabetic status by the abundance of species and MGC in the faecal microbiome. In fact, MGC were found to be better at identifying type 2 diabetes than species, so further work is needed to identify the species of these key MGC.

This gut metagenome model could also be used to classify and perhaps even predict the risk of type 2 diabetes. Based on faecal microbiota analysis, women with impaired glucose tolerance were stratified as having a profile indicating normal glucose tolerance or type 2 diabetes. Those who were predicted to develop type 2 diabetes had higher plasma levels of TAG and C-peptide. When the Chinese data^(^ ^69^ ^)^ were similarly analysed, this revealed a difference in the MGC that discriminated for type 2 diabetes; however, they were not the same as those identified in the Swedish study. This highlights the need to investigate populations from different parts of the world.

Although these results provide further confirmation that the gut microbiota is altered in people with type 2 diabetes, they do not show whether the differences are a consequence, a contributor to or a cause of the disease. Mechanistic studies that might help provide an answer are difficult to conduct in human subjects, so information has been gleaned from animal studies. Germ-free mice display reduced adiposity and are resistant to diet-induced obesity. Professor Bäckhed's group carried out their investigation on inflammatory markers in germ-free mice, those conventionally reared and those colonised with *Escherichia coli*. The presence of a gut microbiota was associated with an impaired glucose metabolism, an increased weight and an abundance of crown-like structures in white adipose tissue. The latter are formed by accumulations of macrophages around dead adipose tissue, and have been associated with obesity. These results indicate that the gut microbiota may contribute to metabolic disease by fuelling inflammation in adipose tissue^(^ ^72^ ^)^.

Another area of research is the role of gut hormones in glucose homeostasis, and how this is affected by the gut microbiota. L cells are glucagon-synthesising endocrine cells found mainly in the distal ileum and colon that are able to sense different nutrients in the gut. GLP-1 has many effects on host physiology, including the promotion of insulin biosynthesis, insulin secretion and islet β-cell survival. It further regulates glucose homeostasis by decreasing glucagon secretion and gastric emptying, and increasing satiety^(^ ^73^ ^)^. Studies with germ-free and conventional mice have shown that the gut microbiota suppresses proglucagon expression and circulating GLP-1 levels through its production of SCFA, which affects the glucose metabolism rate and the intestinal transit time.

Bile acids are detergent molecules synthesised from cholesterol by the liver, which are further metabolised by the gut microbiota into secondary bile acids. Their main function is to solubilise and absorb cholesterol, fat-soluble vitamins and lipids from the intestines, and their synthesis is controlled via the activation of the nuclear receptor farnesoid X receptor in the ileum and liver^(^ ^74^ ^,^ ^75^ ^)^. It is now realised that bile acids are important signalling molecules involved in the regulation of biosynthetic and metabolic pathways in the gut and liver^(^ ^76^ ^)^. Studies comparing germ-free mice with conventionally reared mice showed that the gut microbiota also inhibits bile acid synthesis in the liver by reducing the levels of tauro-β-muricholic acid. The latter is a naturally occurring antagonist to the farnesoid X receptor in the ileum^(^ ^75^ ^)^. Further mice studies have shown that the presence of the gut microbiota induces obesity by a mechanism dependent on the presence of the farnesoid X receptor.

While there has been an explosion of research into the influence of the gut microbiota on obesity and obesity-related disorders, results from animal studies have not always agreed with those from human studies. However, investigations need to continue along both routes so that a clearer picture will emerge, and dietary interventions have more chance of succeeding.

## The gut microbiota and the host: from functionality to disease

### Gut barrier function

The gut barrier is a huge mucosal surface where billions of bacteria interconnect with the largest immune system in the body. It needs to be in harmony with the commensal microbiota and to allow the exchange of molecules and absorption, which means the barrier must be both tight and loose, and this can only be achieved through balanced controlled mechanisms. It was the opinion of Professor Bischoff that studying gut barrier function may help fill the gaps in our understanding of the association between the gut microbiota and disease risks; however, to do this, the gut barrier components and function, as well as its interactions with the intestinal microbiota and other luminal contents, must be better understood.

The intestinal barrier is a functional entity separating the gut lumen from the inner host, and comprising elements that are mechanical (mucus, epithelial layer), humoral (defensins, IgA), cellular or cell-mediated (lymphocytes, innate immune cells), muscular and neurological. Intestinal permeability is a functional feature of this barrier at given sites. Pathologically altered intestinal permeability is one that is non-transiently changed from the normal condition, leading to a loss of intestinal homeostasis, functional impairments and disease risks.

The gut barrier is influenced by exogenous factors, such as infections, toxins, stress, diet, vitamins, pro- and prebiotics, antibiotics and exercise. The effect of exercise was shown in a study of healthy men undergoing a strenuous cycling regimen, which resulted in splanchnic hypoperfusion, small-intestinal injury and transiently increased small-intestinal permeability; all these factors indicated gut barrier dysfunction^(^ ^77^ ^)^. Endogenous factors also regulate the gut barrier, including defensins, cytokines, inflammatory mediators, serotonin, histamine, proteases, neuronal factors, perfusion/oxygen delivery, mucus quality and the cannabinoid system^(^ ^61^ ^,^ ^78^ ^)^. Although many methods and markers are used to assess the integrity of gut barrier function, the normal ranges and the interrelationship of the means are poorly defined^(^ ^79^ ^–^ ^81^ ^)^.

Alterations of the gut barrier have been identified as a key event in the pathogenesis of many diseases^(^ ^82^ ^)^, including intestinal disorders (infectious diarrhoea, IBD, irritable bowel syndrome, ischaemia of the gut) and extra-intestinal diseases (allergies, respiratory infections, chronic inflammatory illness, obesity and metabolic diseases). The causes are not always known but may include nutritional factors, infections and toxins, lack of exposure to microbes in early childhood, and impaired function and diversity of the gut microbiota.

While an altered gut microbiota has been linked to obesity-related disease^(^ ^83^ ^)^, Professor Bischoff believed that more evidence is needed to prove a causal relationship^(^ ^84^ ^–^ ^86^ ^)^. For example, an observational study of severely obese subjects found plasma citrulline and intestinal fatty acid-binding protein levels (markers of gut barrier integrity) were significantly elevated in individuals with chronic hyperglycaemia. This was associated with increased small-intestinal enterocyte mass and increased enterocyte loss^(^ ^87^ ^)^. Research by Professor Bischoff's group has also shown that modulation of the gut barrier is associated with a change to a Western-style diet (personal communication, Professor S Bischoff).

The events leading to gut-barrier associated metabolic liver disease is thought to be as follows: an unhealthy diet (high in fat and fructose, and low in fibre) that leads to impaired gut barrier function and therefore translocation of endotoxin (i.e. lipopolysaccharide) into the host^(^ ^54^ ^)^, triggering low-grade inflammation and then disease (e.g. non-alcoholic fatty liver disease and insulin resistance). These new pathophysiological insights open up the possibility of novel therapeutic interventions, and there has been probiotic research in this area. A study with *Lactobacillus casei* Shirota showed that the probiotic induced a protective effect in a mouse model for fructose-induced liver steatosis, with possible mechanisms of activity involving attenuation of the Toll-like receptor (TLR)-4 signalling cascade in the liver^(^ ^88^ ^)^.

### Recognition of pathogenic bacteria

Invasion by pathogenic bacteria is potentially life-threatening for the susceptible host, thus all its defensive weaponry is mustered. While a key component of this defence is the immune system, the clinical course and eventual outcome of infection does not solely depend on the interaction between the pathogen and the immune system. For example, in GI infections, the commensal microbiota plays a crucial role in both modulating the host immune response and directly competing with the invading micro-organism. Professor Frick started by asking: what makes a bacterium pathogenic, since bacteria can have both commensal and pathogenic traits? Pathogens rapidly adapt to their environment by means of horizontal gene transfer via pathogenicity islands^(^ ^89^ ^)^. These mobile genetic elements have also been found in non-pathogenic species, as they are important for their evolution and adaptation. The pathogenicity islands of *E. coli* have been extensively studied, leading to the realisation that enterohaemorrhagic *E. coli* has evolved from commensal non-pathogenic strains by acquisition of virulence genes coding for Shiga toxin^(^ ^90^ ^)^. Comparative genomics has revealed that *Shigella* actually belong to the *E. coli* species. Both enteroinvasive *E. coli* and *Shigella* have emerged via convergent evolution from other *E. coli* strains, by acquiring virulence factors that enable them to invade the host and cause illness^(^ ^91^ ^)^.

Such pathogenic traits, however, are not the sole reason why symptoms develop after infection. The response of the host also plays a part, as shown, for example, by the signs and symptoms resulting from an inflammatory response. Micro-organisms are recognised by the host by means of pattern recognition receptors such as the TLR, which distinguish friend from foe by means of pathogen-associated molecular patterns on the bacterial surface^(^ ^92^ ^)^. These activate signalling pathways, triggering a defensive immune response^(^ ^93^ ^)^ either directly via the pattern recognition receptors or indirectly by an antigen-specific response, mediated via T cells and antibodies^(^ ^94^ ^)^. *Salmonella* is a classic example to illustrate the infection process of an enteric pathogen^(^ ^95^ ^)^: it is able to subvert the host's immune response by secreting the protein SseI into dendritic cells (DC), which prevents the normal migration of these DC to lymphoid tissues and inhibits the adaptive immune response^(^ ^96^ ^)^. The pathogen can then persist in the body and infect many organs, yet the host remains asymptomatic and is a risk to others because carriers shed high counts of *Salmonella* in their faeces.

The dense, complex microbial community in the gut has many mechanisms that help the host: supporting epithelial cell metabolism; stimulating the mucosa-associated immune system, regulating intestinal angiogenesis; supporting intestinal peristalsis; preventing bacterial overgrowth; destroying enteric toxins; resisting colonisation by pathogens^(^ ^97^ ^)^. The commensal microbiota helps maintain homeostasis through mechanisms such as the regulation of enterocyte and Paneth cell secretion of antimicrobial peptides (e.g. defensins, cathelicidins) that are found in the inner mucus layer. If a pathogen breaks through the mucus layer, there is loss of homeostasis and disruption of gut barrier integrity^(^ ^98^ ^)^.

Professor Frick discussed the problem of patients becoming colonised with commensal species that have acquired antibiotic-resistant genes. Enterococci, for example, are commonly found in the GI tract and are opportunistic pathogens that have adapted well to the hospital environment. The genus includes strains that have become resistant to most antibiotics, including vancomycin. If such strains translocate from the gut, clinicians are running out of options for antibiotics that will work. Finally, Professor Frick asked ‘who’ is responsible for infectious disease – the pathogen; the host; and/or the commensal microbiota? This could be an important consideration when choosing treatment options^(^ ^99^ ^)^.

### Influence of the intestinal microbiota on mucosal immune response: tolerance or defence

Professor Kiyono explained that the GI tract is covered by a single layer of mucosal epithelial cells constantly exposed to antigenic challenges from both pathogenic and commensal micro-organisms. The mucosal immune system is the first line of surveillance and protection against invasion by undesired antigens including pathogens, while tolerant of dietary antigens and the resident beneficial microbiota. Antigens can be transported from the GI lumen across the intestinal epithelial cell wall, for example, via M cells, which are found in the follicle-associated epithelium of Peyer's patches (PP) and the villous epithelium in the small intestine.

Professor Kiyono's group has been characterising M cells, and has profiled the gene expression of M cells from PP, villous-like M cells and intestinal epithelial cells^(^ ^100^ ^)^, and shown that the mucosal immune response in M cells can be initiated by means of a glycoprotein 2-dependent transcytosis pathway^(^ ^101^ ^)^. Recent new findings were that genes for Spi-B transcriptase (*Spib*), uromodulin (*Umod*) and fucosyltransferase 1 (*Fut1*) are specifically expressed by M cells^(^ ^102^ ^,^ ^103^ ^)^. SpiB^− / −^ mice have fewer M cells but some can still be detected in the PP epithelium, suggesting that there must be a SpiB-independent development pathway. These M cells, however, are unique and typically covered by irregular, short microvilli when compared with neighbouring columnar epithelial cells. To investigate the function of this gene, mice were given an oral challenge with *Salmonella enterica* serovar Typhimurium, and some translocation of the enteric pathogens occurred via the M cells in SpiB-deficient mice. Thus, although it is generally acknowledged that Spib is an important transcription factor for M cell development, another transcription factor must be involved^(^ ^104^ ^,^ ^105^ ^)^.

Professor Kiyono then switched his attention to the influence of the commensal microbiota in determining the immune response, stressing that inflammation can be triggered if the delicate balance between the microbes and the immune system is disrupted. As an illustration of how the gut microbiota can protect the host, he described how koalas eat leaves of the Eucalyptus trees that contain cyanide compounds without any harm, possibly because the compounds are degraded by *Pseudomonas* spp. present in the animals' gut. The key role of the GI microbiota in the development of the mucosal immune system was demonstrated in the late 1970s and 1980s in studies with germ-free mice which did develop PP, but they were very small. If *E. coli* or lipopolysaccharide was introduced orally, then PP reached normal maturation and IgA-producing B cells increased, and oral tolerance was induced^(^ ^106^ ^)^.

Further information has come from investigations of gnotobiotic mice colonised with segmented filamentous bacteria and/or clostridia^(^ ^107^ ^)^. Together, these bacteria promoted the development of intraepithelial lymphocytes and IgA-producing cells in the small intestine and intraepithelial lymphocytes only in the colon. Although the dome epithelium of PP is covered with segmented filamentous bacteria, these bacteria were not seen inside; instead, it was found that commensal species such as *Alcaligenes* cohabited in the PP and isolated lymphoid follicles, leading to preferential induction of antigen-specific IgA in the GI tract^(^ ^108^ ^)^. Although only a few cases have been investigated so far, lower levels of *Alcaligenes* have been observed in samples from patients with Crohn's disease. SpiB-negative M cells take up *Alcaligenes* but at a reduced level. In collaboration with Dr David Artis and colleagues, Professor Kiyono's group has shown that depletion of intestinal innate lymphoid cells resulted in peripheral dissemination of *Alcaligenes* spp. This caused a systemic inflammation that could be prevented by administration of IL-22. These experiments indicate that innate lymphoid cells play a critical role in the containment of *Alcaligenes* in the PP^(^ ^109^ ^)^.

The effects of fucosylation of epithelial cells have also been investigated, as glycosylation in general is important for host defences and provides an ecological niche for the commensal microbiota. The *FUT2* gene has been implicated in susceptibility to Crohn's disease^(^ ^110^ ^,^ ^111^ ^)^, and there are also indications that the commensal microbiota (e.g. *Bacteroides thetaiotaomicron*) influences fucose availability in the GI tract^(^ ^112^ ^,^ ^113^ ^)^. Professor Kiyono's group investigated glycosylation in different areas of the GI tract and how this is influenced by the gut bacteria. No glycosylation was observed in germ-free mice, and administration of antibiotics reduced the levels of glycosylation in conventional mice. Segmented filamentous bacteria not only generated T helper 17cells, but also increased intraepithelial lymphocytes and secretory IgA levels, as well as induced glycosylation of intestinal epithelial cells. Currently, the group is investigating how fucosylation of intestinal epithelial cells is regulated.

### Faecalibacterium prausnitzii: a possible role for therapeutic intervention in inflammatory bowel disease

Different lines of evidence indicate that the combined effects of the intestinal microbiota, host genetic and environmental factors lead to an abnormal interaction between the host cells and microbes, resulting in the inflammation observed in IBD^(^ ^114^ ^)^. Professor Wells explained that culture-independent comparative studies of the intestinal microbiota of IBD patients and healthy controls have typically shown that IBD is associated with a decrease in the abundance and biodiversity of Firmicutes and Bacteroidetes phyla, and a corresponding increase in Proteobacteria^(^ ^115^ ^,^ ^116^ ^)^. Significantly, the phylum Firmicutes contains several butyrate-producing species, and the Proteobacteria phylum contains the *E. coli* pathobiome, which has been associated with inflammation.

Investigations of the mucosa-associated microbiota of patients with Crohn's disease by the analysis of resected ileal mucosa have shown that the recurrence of the disease after 6 months was associated with a lower proportion of *F. prausnitzii* (a major member of the Firmicutes phylum). *F. prausnitzii* is one of the most abundant species in human faeces and a major supplier of butyrate to colonic epithelial cells^(^ ^117^ ^)^. This strict anaerobe adheres to the intestinal mucosa even though oxygen is present by diffusion from the underlying intestinal epithelial cells. Khan *et al.* ^(^ ^25^ ^)^ explained this apparent paradox by showing that *F. prausnitzii* can use an extracellular electron shuttle of flavins and thiols (compounds present in the human gut) to transfer electrons to oxygen, which allows the bacteria to grow at the interface of oxic/anoxic conditions. This oxygen-transfer system allows *F. prausnitzii* to grow in the loose mucus layer, at a depth where the gradient of oxygen offers the species a unique ecological niche in the gut.

Animal and human studies have indicated that normally bacteria do not penetrate the inner mucus layer in the colon, but this may not be the case in IBD. Penetration of the mucus layer was observed in animal models that spontaneously developed colitis, as well as in patients with active UC where bacteria were observed to reach the intestinal epithelium^(^ ^118^ ^,^ ^119^ ^)^.

Professor Well's group recently found that a strain of *F. prausnitzii* (HTF-F) can produce an extracellular polymeric matrix (EPM), which is involved in biofilm formation in the gut at the interface between the firm mucus and the loose mucus. Electron microscopy of the bacterial surface of this strain revealed that it appeared to consist of three layers, and there were some unusual structures visible on the outer wall. Comparison of the anti-inflammatory capabilities of *F. prausnitzii* with other commensal bacteria showed that *F. prausnitzii* tended to induce IL-10 in peripheral blood mononuclear cells, whereas a *Lactobacillus plantarum* strain induced IL-12p70. Similar trends were observed when human DC were stimulated with two *F. prausnitzii* strains (A2-165 and HTF-F). *F. prausnitzii* can differentially affect T-cell activation and polarisation^(^ ^120^ ^)^, as was shown by Professor Well's group *in vitro* using a transgenic ovalbumin-specific T-cell transfer model. *F. prausnitzii* A2-165 induced the proliferation of CD4^+^ ovalbumin-specific T cells, and decreased the percentage of interferon-γ-positive T cells and activated/proliferating T cells. The EPM alone did not activate immune cells *in vitro*, but there was a TLR2-dependent immunomodulatory effect on cytokine responses to *L. plantarum* in human and murine DC.

In the murine model of UC, *F. prausnitzii* strains and the EPM alone were able to attenuate clinical symptoms, but to differing degrees: the EPM-forming strain (HTF-F) had a greater effect than the non-EPM-forming strain (A2-165), and both were more effective than the EPM alone. The control mice developed colitis and lost weight, but all the bacteria-treated mice showed protective effects: reduction of weight loss; reduction of symptoms; increase in colon length. The greater effect of the strain HTF-F may be due to the combination of its immunomodulatory abilities and its EPM, but the precise anti-inflammatory mechanism of the EPM awaits further identification of the active component. Professor Wells concluded that these results suggest that *F. prausnitzii* and the EPM may have potential application in the treatment of IBD.

### Preclinical probiotic studies in IBD: consideration of the gut mucus layer

Professor Rescigno explained that in the intestine, DC are found in the lamina propria (LP) of the villi, in the mesenteric lymph nodes, lymphoid aggregates and PP. Probably the greatest number of antigen-presenting cells in the gut is found in the LP, outnumbering those in the mesenteric lymph nodes or PP. Based on functionality, the DC found in the LP of mice can be divided into subgroups depending on whether they express CX3CR1 (the receptor of the chemokine fracktalkine) and CD103 (the receptor for the epithelial cell adhesion molecule E cadherin). CX3CR1^+^ DC extend protrusions from the LP across the tight junctions of the intestinal epithelial cells to interact with the contents of the gut lumen, capturing bacteria there. This is done without compromising the epithelial barrier because the DC can express tight junction proteins^(^ ^121^ ^)^. Dynamic imaging using analysis of CD11c-enhanced green fluorescent protein (EFGP) or major histocompatibility complex CII-EGFP mice has given visual confirmation of DC extending into the small bowel, showing that this happens frequently in the proximal jejunum but much less in the terminal ileum^(^ ^122^ ^)^. These DC are somewhat similar to macrophages as they are sessile and remain in the gut.

In contrast, CD103^+^ DC can migrate into the draining mesenteric lymph nodes where they drive the conversion of Foxp3^+^ regulatory T cells. CD103^+^ conventional DC enter the gut as progenitors, becoming tolerogenic via their interaction with the local microenvironment and, in particular, with the intestinal epithelial cells. Human intestinal epithelial cells drive the development of anti-inflammatory DC by releasing thymic stromal lymphopoietin, which inhibits IL-12 production by DC if phenotypically activated by bacteria, polarising T cells towards a mucosal non-inflammatory T helper 2 cell phenotype or regulatory T cells. CX3CR1^+^ cells are able to take up bacteria and food antigens that they then transfer to CD103^+^ DC. This interaction allows the establishment of tolerance to luminal antigens. Other factors such as retinoic acid and transforming growth factor-β are also thought to be important in the induction of homeostasis in the gut.

While the mucus layer is thinner in the duodenum to enable DC to extend into the gut lumen, it is thicker in the ileum. However, the mucus layer is thinner in IBD patients, who also have more mucosa-associated bacteria. Professor Rescigno discussed an *ex vivo* organ model system developed by her group, which involves a human mucosa explant onto which a cylinder is applied, without damaging the cells, to maintain the apical to basolateral polarity of the tissue. This has been used to study the effect of applying bacteria, including probiotics, to the apical surface, in order to mimic their interactions with immune cells through the mucus and epithelial cell layers in the gut. The activity of three *Lactobacillus* probiotics and a *Salmonella* strain was investigated, with differing results. No significant change in the healthy condition of the mucosa tissue and the normal profile of secreted cytokines was observed after the mucosa was incubated with *Lactobacillus paracasei* B21060 or *Lactobacillus rhamnosus* GG, but incubation with *L. plantarum* NCIMB8826 caused tissue deterioration. When mucosa from IBD patients was used in the model, all the three strains caused alterations in the tissue structure. The group then examined the potential anti-inflammatory activity of soluble metabolic products of the probiotics (termed postbiotics)^(^ ^123^ ^,^ ^124^ ^)^. *Salmonella*-induced tissue damage in the organ model was prevented by a culture supernatant from the *L. paracasei* strain. The supernatant also reduced the aggravated inflammation caused by the probiotic strains in inflamed tissue samples^(^ ^125^ ^)^.

Professor Rescigno believes that these data indicate that, even though certain strains have been shown to help prolong IBD remission periods, preclinical studies are necessary before use of any probiotics in IBD patients with active disease^(^ ^126^ ^)^. She suggested that anti-inflammatory postbiotics might be a valid alternative for treatment.

### Clinical observations: modulation of the gut microbiota in inflammatory bowel disease patients

Various lines of evidence have implicated the intestinal microbiota as a driver of inflammation in IBD, observed Dr Hart: for example, diversion of the faecal stream (rich in bacteria) ameliorated inflammation in patients with Crohn's disease, whereas reintroduction of the ileal contents to the diverted bowel induced inflammation, and reduced diversity of the faecal microbiota was observed in patients with Crohn's disease^(^ ^37^ ^)^. A prospective study at St Mark's Hospital found a significant decrease in the diversity and richness of the colonic microbiota in UC patients during remission, which decreased further during clinical relapse, with the loss of normal taxa such as *Bacteroides*, *Escherichia*, *Eubacterium*, *Lactobacillus* and *Ruminococcus* spp.^(^ ^127^ ^)^. Reduced diversity of the faecal microbiota has also been observed in pouchitis patients^(^ ^128^ ^)^; a significant increase in Proteobacteria and decreases in Bacteroidetes and *F. prausnitzii* have also been shown in UC patients.

Reduced numbers of *F. prausnitzii* have been observed previously in patients with Crohn's disease^(^ ^43^ ^)^, but clinical correlation has yet to be proved, as was highlighted by a recent UK study. Culture-independent analysis of the colonic mucosa, which showed reduced microbial diversity in children with Crohn's disease but not with UC, found higher levels of *F. prausnitzii* in patients with Crohn's disease compared with healthy controls^(^ ^129^ ^)^. Recent analysis of intestinal biopsies and faecal samples from 231 IBD patients and healthy controls also showed differences in microbial function: major shifts in oxidative stress pathways; decreased carbohydrate metabolism; decreased amino acid synthesis. In ileal Crohn's disease, there were notable increases in virulence and secretion pathways^(^ ^130^ ^)^.

A number of treatment options targeting the microbiota have become well established in IBD, including antibiotics and probiotics. Up to half of the patients undergoing pouch surgery for UC develop pouchitis; 5–10 % of these may get truly refractory disease. Pouchitis is always treated with antibiotics: a clinical protocol developed at St Mark's Hospital uses metronidazole or ciprofloxacin in the first instance, followed by both, and then a targeted antibiotic as necessary. After the patient responds, a probiotic such as VSL#3 is given to maintain remission. In one study evaluating antibiotics for the treatment of perianal fistulas in patients with Crohn's disease, a trend for better remission and response was observed with the use of ciprofloxacin^(^ ^131^ ^)^. Post-operative metronidazole treatment for 3 months can also decrease the severity of early recurrence of Crohn's disease following ileal resection^(^ ^132^ ^)^.

Dr Hart outlined the main challenges in IBD research: patient heterogeneity; multiple possible confounders; difficulty in defining ‘healthy’ controls; what, when and how to sample (faeces or mucosa). She advised taking samples over a period of time rather than multiple samples at the same time from the same region, keeping good communication between the diverse staff involved, and choosing the right methodology. Several probiotic studies in IBD have been conducted using different strains (single and mixtures). Promising results are emerging for UC but very little for Crohn's disease. The largest UC study to date, lasting 12 months and involving 327 patients given either *E. coli* Nissle 1917 or mesalazine (500 mg three times daily), found the probiotic as effective as the standard drug treatment in maintaining remission^(^ ^133^ ^)^. VSL#3 (a multi-strain powder) has also shown beneficial effects^(^ ^134^ ^,^ ^135^ ^)^. Dr Hart also cited a trial investigating fructo-oligosaccharides in active Crohn's disease. The prebiotic showed no beneficial effects, and there was no difference in the faecal levels of *F. prausnitzii*, although some evidence of the modulation of DC function did exist^(^ ^136^ ^)^. However, this does not necessarily mean that prebiotics may not have a role in maintaining remission or preventing disease onset in individuals at high risk.

Probiotic studies need a proof of principle, argued Dr Hart, which considers both disease pathogenesis and mechanism of activity. Many issues remain unresolved: choice of strain and dosage; duration of treatment; use of concomitant treatment; clinical and genetic subsets; potential synergies or antagonism between strains. Research at St Mark's Hospital, which has focused on immune modulation and how probiotics influence DC, has shown that effects are strain/product-specific and that probiotics influence the immune response at an early stage (antigen presentation by DC), with indications of possible benefits^(^ ^137^ ^)^. These have been confirmed in *ex vivo* studies. For example, VSL#3 and corticosteroid treatment of rectal biopsy samples from UC patients induced apparently improved intestinal DC function, increased regulatory cytokines, and reduced pro-inflammatory cytokines and TLR expression^(^ ^138^ ^)^. Studies in a murine experimental colitis model administered with VSL#3 indicated that bacterial DNA was responsible for the protective effects and that TLR9 (the receptor recognising bacterial DNA) signalling was essential for the anti-inflammatory effect^(^ ^139^ ^)^. Studies with isolated DC and VSL#3 showed that bacterial DNA induced an immunoregulatory cytokine profile^(^ ^140^ ^)^. Furthermore, an extracellular, soluble protein secreted by a *L. plantarum* strain has been identified, which is resistant to proteolysis and promotes the production of regulatory IL-10 in intestinal DC from healthy people. T cells stimulated by these DC cells had an immunoregulatory and skin-homing profile^(^ ^141^ ^)^.

Faecal transplantation (another form of microbiota modulation) has been used to treat fulminant and refractory *C. difficile* infection^(^ ^142^ ^)^. In fact, the first randomised controlled trial was stopped after interim analysis showed *C. difficile*-associated diarrhoea resolved in 81 % of patients after the first faecal infusion^(^ ^143^ ^)^. After treatment, increased diversity of the faecal bacteria was observed, becoming more similar to that of healthy donors. A mouse model of *C. difficile* infection has also been used to develop a mixture of six phylogenetically diverse intestinal bacteria, which re-established a healthy intestinal microbiota and cleared infection in mice^(^ ^144^ ^)^. These successes have prompted IBD investigations^(^ ^145^ ^)^. A systematic review in 2012^(^ ^146^ ^)^ identified seventeen trials involving faecal microbiota transplant (none controlled) in a total of forty-one IBD patients, with a follow-up period of 2 weeks to 13 years, with administration via colonoscopy/enema or enteral tube. The majority (*n* 19/25) had improved symptoms, ceased IBD medications (*n* 13/17) and achieved disease remission (*n* 15/24). It was concluded that, while the evidence was limited and weak, it did indicate potential if standard treatments were unsuccessful. However, a recent pilot study investigating faecal transplantation for chronic refractory pouchitis has found that nasogastric administration did not achieve clinical remission but ciprofloxacin sensitivity was regained in two patients with extended-spectrum β-lactamases-producing coliforms, enabling ciprofloxacin to then be used for maintenance^(^ ^147^ ^)^. Dr Hart noted many unresolved issues regarding faecal transplantation for IBD, including establishment of safety; however, clinicians and patients remain interested in this treatment.

### Coeliac disease

Coeliac disease is an autoimmune disorder, mainly triggered by dietary gluten in the genetically susceptible. Professor Sanz explained that intake of wheat gluten (or similar proteins found in rye and barley) activates an inflammatory T helper 1 response resulting in severe injury to the tissue of the small intestine and eventually malabsorption syndrome. To avoid illness, sufferers adhere strictly to a gluten-free diet.

The disease is strongly associated with carriage of human leucocyte antigen (HLA)-DQ genes with most sufferers carrying a variant of DQ2 or DQ8. (HLA-DQ genes code for proteins involved in antigen-recognition). It is still not understood, however, why only a small percentage of people with these genes become ill, thus environmental triggers may also be involved. The increasing diagnosis in adulthood further indicates that introduction of gluten into the diet is not the only environmental trigger. Attention has recently shifted to the possible role of the intestinal microbiota. Human studies have indicated that environmental exposures affecting the initial microbial colonisation of babies may be risk factors for disease development. Breast-feeding, which promotes a protective microbiota in neonates^(^ ^148^ ^)^, may help protect against disease development particularly if done when gluten is introduced for the first time^(^ ^149^ ^)^. It is not yet clear, though, whether breast-feeding delays disease onset or gives permanent protection.

Delivery mode also affects the acquisition and structure of the initial microbiota, as has been noted earlier. Vaginally delivered babies acquire a microbial profile similar to that of the mother's vagina (predominance of *Lactobacillus*, *Prevotella* or *Sneathia* spp.), whereas babies born by caesarean section have a profile similar to that of the maternal skin (predominance of *Staphylococcus*, *Corynebacterium* or *Propionibacterium* spp.)^(^ ^3^ ^)^. A case–control study in Sweden reported a correlation between elective caesarean delivery and later onset of coeliac disease^(^ ^150^ ^)^. Infections and antibiotic exposure, which also affect the intestinal microbiota, may also be risk factors^(^ ^151^ ^,^ ^152^ ^)^.

Professor Sanz's group analysed the duodenal microbiota of children with coeliac disease, and found that *Bacteroides* and *E. coli* groups were significantly more abundant in those with active disease compared with healthy controls or symptom-free patients. The ratio of *Lactobacillus*–*Bifidobacterium*: *Bacteroides*–*E. coli* was significantly lower compared with the ratio for those who were healthy^(^ ^153^ ^)^. A later study has confirmed that the duodenal and faecal microbiota was unbalanced in children with untreated disease, and only partially restored after a long period of eating a gluten-free diet^(^ ^154^ ^)^. A difference in the profile of *Bacteroides* spp. has also been observed in the intestinal microbiota of patients (both with active and inactive disease after adherence to a gluten-free diet), with greater abundance of *Bacteroides fragilis* strains with metalloprotease activities and reduced levels of *Bacteroides ovatus*. (Metalloproteases are virulence factors: enterotoxins associated with diarrhoea in human subjects and associated with alterations of tight junctions and inflammation in a murine model.)^(^ ^155^ ^,^ ^156^ ^)^ Increased numbers of staphylococci and enterobacteria have been shown in patients with active disease, with numbers restored after adherence to a gluten-free diet^(^ ^157^ ^,^ ^158^ ^)^, but an increased abundance of *Staphylococcus epidermidis* strains carrying a methicillin-resistance gene has been observed in patients with active and inactive disease^(^ ^159^ ^)^. Furthermore, virulent clones of *E. coli* harboured increased virulence factors, for example, haemolysin, P fimbriae and capsule K5, making them more successful as pathogens. Faecal bifidobacteria were also reduced in patients with active and inactive disease.

Evidence of dysbiosis in coeliac disease is supported by animal studies. Fragments of gliadin (a dietary wheat gluten protein), alone or in combination with interferon-γ, decreased the number of goblet cells in ligated ileal loops taken from germ-free rats. (Goblet cells produce mucus, which forms the outermost layer of the gut mucosa.) This was more pronounced in the presence of *E. coli* and *Shigella*. Goblet cell numbers in the small intestine were restored if *B. bifidum* CLCT7365 was co-incubated; this also resulted in increased production of chemotactic factors and inhibitors of metalloproteases. The decline in goblet cell numbers observed with Gram-negative strains was accompanied by a significant increase in mucin secretion, thus it was postulated that these were related: excessive mucin production exhausted the cells and caused changes in the architecture of the epithelial layer (restored by bifidobacteria). The enterobacteria strains caused damage to the tight junction, increasing gliadin translocation into the LP^(^ ^160^ ^)^.

Some studies have not implicated intestinal dysbiosis with disease; for example, a study in The Netherlands using a 16S–23S interspacer region-based method has shown that microbiome diversity and composition of small-bowel biopsies from children were similar regardless of whether or not they had coeliac disease^(^ ^161^ ^)^. Dysbiosis has been reported in other studies, such as the one in Sweden using 16S ribosomal DNA sequencing, culture and electron microscopy to analyse small-intestinal biopsies, which found the normal mucosal-associated microbiota in the proximal small intestine to be limited in children with disease. Scanning electron micrographs of biopsies showed significant enrichment of rod-shaped bacteria, thought to be *Clostridium*, *Prevotella* and *Actinomyces*. The biopsies were taken from children born during a period when Sweden experienced an epidemic of new coeliac disease cases^(^ ^162^ ^)^.

Furthermore, two Italian groups have observed a peculiar intestinal microbiota profile in children with disease: one showed differences in diversity with greater prevalence of *Bacteroides vulgatus* and *E. coli* ^(^ ^163^ ^)^ and the other showed lower faecal lactobacilli and bifidobacteria with higher levels of *Bacteroides*, *Staphylococcus* and certain Enterobacteriaceae spp.^(^ ^164^ ^)^. A study in Finland went further, relating GI symptoms of adult patients to changes in their microbiota composition, including lower biodiversity^(^ ^157^ ^)^.

Finally, Professor Sanz described the PROFICEL project: a prospective 3-year study following a cohort of 164 babies with a first-degree relative affected by coeliac disease, examining whether they carry the human leucocyte antigen-DQ gene and the risk of developing disease^(^ ^158^ ^)^, as well as clinical, dietary, immunological and faecal microbial parameters (at 7 d, 1 and 4 months of age)^(^ ^165^ ^)^. Initial results showed that, regardless of whether breast- or formula-fed, infants with an increased genetic risk of disease had lower numbers of faecal *Bifidobacterium* spp. and *B. longum*, and higher numbers of *Staphylococcus*, which may indicate that the host's human leucocyte antigen-DQ genotype favours staphylococcal colonisation. In general, breast-feeding appeared to reduce the genotype-related differences in microbiota, which may partly explain the protective role observed with breast-feeding.

To conclude, Professor Sanz suggested that a more provocative theory for coeliac disease could now be considered, recognising the relationship between human leucocyte antigen-DQ genes and the pattern of microbial colonisation in the gut, and gut dysbiosis as a trigger for disease. Antibiotic use and exposure to bacterial or viral pathogens, which can cause gut dysbiosis, have all been linked to an increased risk of coeliac disease^(^ ^166^ ^–^ ^172^ ^)^. The clinical implications of this need to be explored. However, while there may be potential in modulating the gut microbiota via dietary interventions, as yet, there is insufficient evidence to indicate whether this would have any benefit for coeliac disease.

## Conclusions

The breadth of research described during the symposium clearly demonstrated the extent of international interest in the intestinal microbiota and its influence on health status and disease risk. The research described ranged from large population studies, to clinical trials using dietary and other interventions to modulate the microbiota, and mechanistic studies investigating bacterial effects at the cellular and molecular level on the gut-associated immune system, the gut barrier and the gut mucosa.

The speakers gave several recommendations and warnings for the direction of future research. For instance, the knowledge that bifidobacteria strains can be transmitted from mothers to babies born by vaginal delivery but not to babies born by caesarean section indicates that we need to understand more about the microbiota during pregnancy and its potential transfer to the baby, particularly for women with conditions that predispose them to a less healthy microbiota. The strong and rapid influence of change of residence and diet on the gut microbiota of older people appears to correlate with health status and disease risk, indicating a need to investigate whether modulation of the microbiota improves health status in old age.

Changes in macronutrient intake, especially non-digestible carbohydrates, alter the colonic microbial profile: for example, reduced carbohydrate results in lower levels of butyrate and *Roseburia*-related butyrate producers. Certain species, such as *R. bromii*, have been identified as a keystone species in the colon. We need to understand the health implications if such species are absent, and whether these key species can, or should be, reintroduced. Further work is needed to elucidate the complex cross-feeding between species in the gut, and how this differs between individuals according to the lifestyle and diet.

Metagenomic studies have revealed low species diversity and/or dysbiosis in the gut microbiota of people with various diseases, including IBD, obesity-related disorders, diabetes, coeliac disease, allergy, frailty in senior citizens and irritable bowel syndrome. This prompted speculation that reduced microbial diversity could even be a marker of disease risk. It has been suggested that knowledge of a person's microbiome might eventually aid diagnosis and clinical management of patients, but a lot more research is needed, as was highlighted by the discovery that different MGC predicted type 2 diabetes in China and Sweden. Uncertainty remains as to whether changes in the microbiota are a cause or effect of specific diseases, and there is insufficient understanding of the mechanisms involved. In some diseases, for example, there are indications that certain bacteria may act as triggers or drivers of disease while other species may offer benefit. There has sometimes been poor correlation between *in vitro*, animal and human studies; the latter are required to confirm any effects of dietary interventions that modulate the gut microbiota.

The symposium underlined the importance of continuing to acquire scientific knowledge about the influence of the gut microbiota on health, in order to identify targets and interventions to reduce the risk of disease or develop treatments. It is also essential that key findings are translated to the medical community, so that any dietary interventions or risk markers that are identified can be implemented as part of a positive strategy for health maintenance.
